# *N*-(2-Acetyl­phen­yl)benzene­sulfonamide

**DOI:** 10.1107/S1600536809033273

**Published:** 2009-08-26

**Authors:** R. R. Saravanan, V. Dhayalan, A. K. Mohanakrishnan, G. Chakkaravarthi, V. Manivannan

**Affiliations:** a Department of Physics, PRIST University, Thanjavur 614 904, Tamil Nadu, India; b Department of Organic Chemistry, University of Madras, Guindy Campus, Chennai 600 025, India; c Department of Physics, CPCL Polytechnic College, Chennai 600 068, India; d Department of Research and Development, PRIST University, Vallam, Thanjavur 613 403, Tamil Nadu, India

## Abstract

In the title compound, C_14_H_13_NO_3_S, the phenyl ring makes a dihedral angle of 81.5 (1)° with the benzene ring. The mol­ecular structure is stabilized by an intra­molecular N—H⋯O hydrogen bond and weak C—H⋯O inter­actions. In the crystal structure, mol­ecules are linked by weak inter­molecular C—H⋯O and C—H⋯π inter­actions.

## Related literature

For the biological activity of benzene­sulfonamide derivatives, see: Badr (2008 ▶); Hanafy *et al.* (2007 ▶); Yang *et al.* (2002 ▶). For related structures, see: Chakkaravarthi *et al.* (2007 ▶); Li & Yang (2006 ▶). For graph-set notation, see: Bernstein *et al.* (1995 ▶).
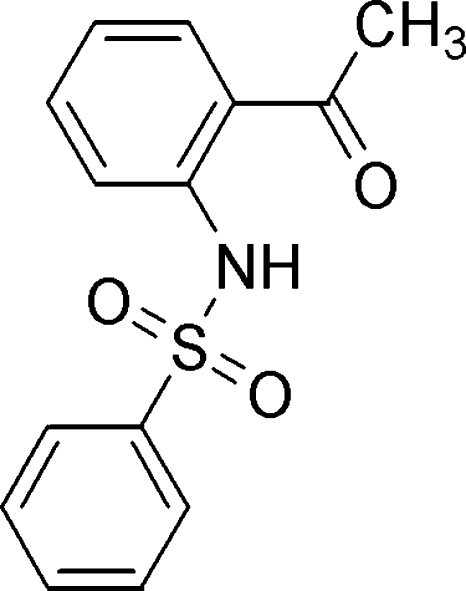


## Experimental

### 

#### Crystal data

C_14_H_13_NO_3_S
*M* *_r_* = 275.31
Triclinic, 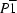

*a* = 7.9909 (3) Å
*b* = 8.6860 (4) Å
*c* = 10.0701 (4) Å
α = 88.016 (2)°
β = 68.673 (3)°
γ = 83.424 (2)°
*V* = 646.79 (5) Å^3^
*Z* = 2
Mo *K*α radiation
μ = 0.25 mm^−1^
*T* = 295 K
0.24 × 0.20 × 0.20 mm

#### Data collection

Bruker Kappa APEX2 diffractometer
Absorption correction: multi-scan (**SADABS**; Sheldrick, 1996 ▶) *T*_min_ = 0.942, *T*_max_ = 0.951
18690 measured reflections
5104 independent reflections
3886 reflections with *I* > 2σ(*I*)
*R*_int_ = 0.023

#### Refinement

*R*[*F* ^2^ > 2σ(*F* ^2^)] = 0.045
*wR*(*F* ^2^) = 0.142
*S* = 1.03
5104 reflections
173 parameters
H-atom parameters constrained
Δρ_max_ = 0.35 e Å^−3^
Δρ_min_ = −0.41 e Å^−3^

### 

Data collection: *APEX2* (Bruker, 2004 ▶); cell refinement: *SAINT* (Bruker, 2004 ▶); data reduction: *SAINT*; program(s) used to solve structure: *SHELXS97* (Sheldrick, 2008 ▶); program(s) used to refine structure: *SHELXL97* (Sheldrick, 2008 ▶); molecular graphics: *PLATON* (Spek, 2009 ▶); software used to prepare material for publication: *SHELXL97*.

## Supplementary Material

Crystal structure: contains datablocks global, I. DOI: 10.1107/S1600536809033273/is2453sup1.cif

Structure factors: contains datablocks I. DOI: 10.1107/S1600536809033273/is2453Isup2.hkl

Additional supplementary materials:  crystallographic information; 3D view; checkCIF report

## Figures and Tables

**Table 1:** Hydrogen-bond geometry (Å, °)

*D*—H⋯*A*	*D*—H	H⋯*A*	*D*⋯*A*	*D*—H⋯*A*
N1—H1⋯O3	0.86	2.03	2.596 (2)	123
C2—H2⋯O1	0.93	2.52	2.893 (2)	104
C12—H12⋯O1	0.93	2.40	3.057 (2)	128
C11—H11⋯O1^i^	0.93	2.51	3.380 (2)	156
C14—H14*C*⋯*Cg*1^ii^	0.96	2.96	3.763 (2)	142

Symmetry codes: (i) 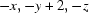; (ii) 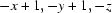. *Cg*1 is the centroid of the benzene C7–C12 ring.

**Fig. 1.**
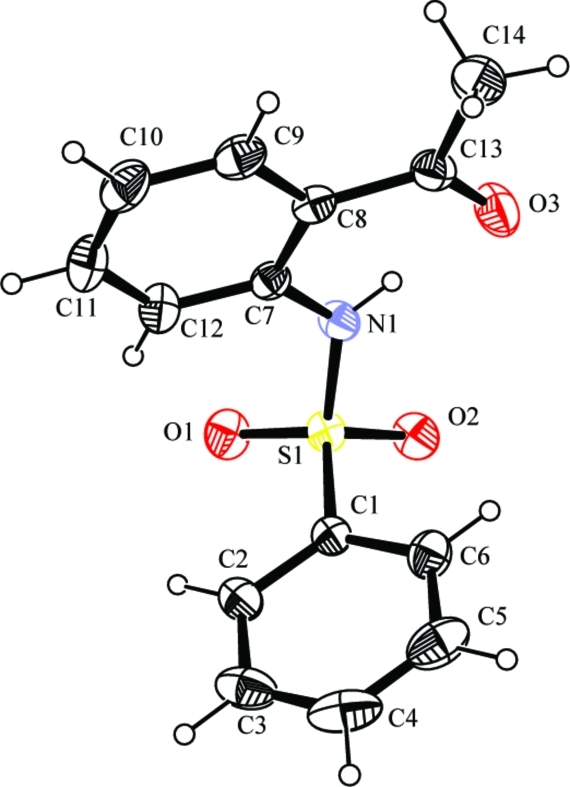
The molecular structure of (I), with atom labels and 30% probability displacement ellipsoids for non-H atoms.

**Fig. 2.**
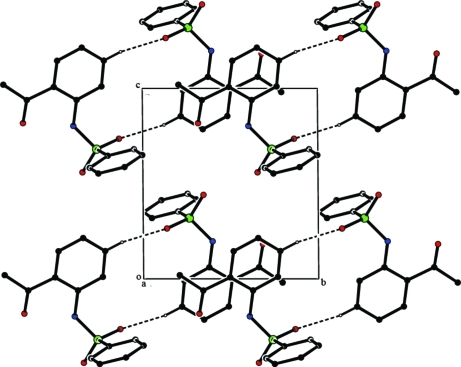
The packing of (I), viewed down the a axis. Hydrogen bonds are shown as dashed lines. H atoms not involved in hydrogen bonding have been omitted.

## supplementary crystallographic information

### Comment

The benzenesulfonamide derivatives are known to exhibit antitumor (Yang *et al.*, 2002), anti-bacterial (Badr, 2008) and anti-fungal (Hanafy *et al.*, 2007) activities. The geometric parameters in (I) (Fig. 1) agree with the reported values of similar structures (Chakkaravarthi *et al.*, 2007; Li & Yang, 2006).

The phenyl ring C1—C6 makes the dihedral angle of 81.5 (1)° with the benzene ring C7—C12. A distorted tetrahedral geometry [O1—S1—N1 109.49 (6)° and O2—S1—N1 104.32 (6)°] is observed around the S1 atom. The molecular structure is stabilized by weak intramolecular C—H···O and N—H···O interactions and the molecules are linked by weak intermolecular C—H···O and C—H···π interactions (Fig. 2 & Table 1). The intramolecular N1—H1···O3 interaction generates a six-membered ring, with graph-set motif S(6) and the intermolecular C11—H11···O1 interaction generates a fourteen membered ring, with graph-set motif *R*_2_^2^(14).

### Experimental

To a stirred solution of 1-(2-aminophenyl)ethanone (3.0 g, 22.19 mmol) in dry DCM (50 ml) at room temperature, pyridine (1.75 g, 22.12 mmol) was slowly added. After 10 min, PhSO_2_Cl (4.71 g, 26.61 mmol) was added and stirred at room temperature for 15 h. Then the reaction mixture was poured over crushed ice containing conc. HCl (10 ml), work up of the reaction followed by recrystallization from CDCl_3_ gave the compound.

### Refinement

H atoms were positioned geometrically and refined using riding model with C—H = 0.93 Å and *U*_iso_(H) = 1.2*U*_eq_(C) for aromatic C—H, N—H = 0.86 Å and *U*_iso_(H) = 1.2*U*_eq_(C) for N—H, and C—H = 0.96 Å and *U*_iso_(H) = 1.5*U*_eq_(C) for CH_3_.

### Crystal data

**Table:**

C_14_H_13_NO_3_S	*Z* = 2
*M**_r_* = 275.31	*F*(000) = 288
Triclinic, *P*1	*D*_x_ = 1.414 Mg m^−^^3^
Hall symbol: -P 1	Mo *K*α radiation, λ = 0.71073 Å
*a* = 7.9909 (3) Å	Cell parameters from 8349 reflections
*b* = 8.6860 (4) Å	θ = 2.2–33.4°
*c* = 10.0701 (4) Å	µ = 0.25 mm^−^^1^
α = 88.016 (2)°	*T* = 295 K
β = 68.673 (3)°	Block, colourless
γ = 83.424 (2)°	0.24 × 0.20 × 0.20 mm
*V* = 646.79 (5) Å^3^	

### Data collection

**Table:**

Bruker Kappa APEX2 diffractometer	5104 independent reflections
Radiation source: fine-focus sealed tube	3886 reflections with *I* > 2σ(*I*)
graphite	*R*_int_ = 0.023
ω and φ scans	θ_max_ = 33.6°, θ_min_ = 2.2°
Absorption correction: multi-scan (*SADABS*; Sheldrick, 1996)	*h* = −12→12
*T*_min_ = 0.942, *T*_max_ = 0.951	*k* = −13→13
18690 measured reflections	*l* = −15→15

### Refinement

**Table:**

Refinement on *F*^2^	Primary atom site location: structure-invariant direct methods
Least-squares matrix: full	Secondary atom site location: difference Fourier map
*R*[*F*^2^ > 2σ(*F*^2^)] = 0.045	Hydrogen site location: inferred from neighbouring sites
*wR*(*F*^2^) = 0.142	H-atom parameters constrained
*S* = 1.03	*w* = 1/[σ^2^(*F*_o_^2^) + (0.0792*P*)^2^ + 0.0784*P*] where *P* = (*F*_o_^2^ + 2*F*_c_^2^)/3
5104 reflections	(Δ/σ)_max_ < 0.001
173 parameters	Δρ_max_ = 0.35 e Å^−^^3^
0 restraints	Δρ_min_ = −0.41 e Å^−^^3^

### Fractional atomic coordinates and isotropic or equivalent isotropic displacement parameters (Å^2^)

**Table:**

	*x*	*y*	*z*	*U*_iso_*/*U*_eq_	
S1	0.21195 (4)	0.73664 (3)	−0.31916 (3)	0.04029 (10)	
O1	0.07767 (14)	0.86458 (13)	−0.26435 (13)	0.0585 (3)	
O2	0.21545 (16)	0.65492 (13)	−0.44094 (11)	0.0551 (3)	
O3	0.3126 (2)	0.31720 (13)	−0.18266 (13)	0.0665 (3)	
N1	0.18906 (16)	0.60455 (13)	−0.19706 (11)	0.0434 (2)	
H1	0.1726	0.5138	−0.2181	0.052*	
C1	0.42453 (17)	0.80008 (14)	−0.35373 (12)	0.0382 (2)	
C2	0.4361 (2)	0.95013 (16)	−0.31914 (15)	0.0486 (3)	
H2	0.3324	1.0179	−0.2762	0.058*	
C3	0.6061 (3)	0.9970 (2)	−0.35014 (18)	0.0661 (5)	
H3	0.6175	1.0975	−0.3282	0.079*	
C4	0.7578 (3)	0.8955 (3)	−0.4131 (2)	0.0755 (6)	
H4	0.8714	0.9279	−0.4334	0.091*	
C5	0.7442 (2)	0.7457 (3)	−0.4469 (2)	0.0717 (5)	
H5	0.8481	0.6780	−0.4893	0.086*	
C6	0.5762 (2)	0.69662 (19)	−0.41754 (16)	0.0528 (3)	
H6	0.5653	0.5963	−0.4401	0.063*	
C7	0.19359 (15)	0.62435 (14)	−0.06059 (12)	0.0378 (2)	
C8	0.24861 (16)	0.49460 (15)	0.00745 (12)	0.0396 (2)	
C9	0.2463 (2)	0.51504 (19)	0.14513 (14)	0.0522 (3)	
H9	0.2818	0.4304	0.1916	0.063*	
C10	0.1932 (2)	0.6560 (2)	0.21426 (16)	0.0603 (4)	
H10	0.1927	0.6662	0.3060	0.072*	
C11	0.1410 (2)	0.7816 (2)	0.14616 (17)	0.0626 (4)	
H11	0.1061	0.8776	0.1920	0.075*	
C12	0.1396 (2)	0.76713 (18)	0.01025 (16)	0.0523 (3)	
H12	0.1025	0.8529	−0.0341	0.063*	
C13	0.30558 (19)	0.34007 (16)	−0.06119 (15)	0.0468 (3)	
C14	0.3558 (3)	0.2063 (2)	0.0194 (2)	0.0679 (5)	
H14A	0.3861	0.1139	−0.0380	0.102*	
H14B	0.2557	0.1924	0.1059	0.102*	
H14C	0.4581	0.2265	0.0420	0.102*	

### Atomic displacement parameters (Å^2^)

**Table:**

	*U*^11^	*U*^22^	*U*^33^	*U*^12^	*U*^13^	*U*^23^
S1	0.04233 (17)	0.04223 (16)	0.04242 (17)	−0.00101 (11)	−0.02377 (13)	0.00144 (11)
O1	0.0481 (5)	0.0583 (6)	0.0696 (7)	0.0125 (4)	−0.0272 (5)	0.0008 (5)
O2	0.0710 (7)	0.0605 (6)	0.0490 (5)	−0.0116 (5)	−0.0384 (5)	0.0009 (4)
O3	0.1044 (10)	0.0446 (5)	0.0568 (6)	−0.0009 (6)	−0.0384 (6)	−0.0043 (4)
N1	0.0550 (6)	0.0410 (5)	0.0411 (5)	−0.0103 (4)	−0.0243 (5)	0.0022 (4)
C1	0.0429 (6)	0.0398 (5)	0.0355 (5)	−0.0036 (4)	−0.0189 (4)	0.0031 (4)
C2	0.0650 (8)	0.0437 (6)	0.0444 (6)	−0.0105 (6)	−0.0276 (6)	0.0039 (5)
C3	0.0859 (12)	0.0725 (10)	0.0590 (9)	−0.0414 (10)	−0.0410 (9)	0.0211 (8)
C4	0.0614 (10)	0.1176 (17)	0.0625 (10)	−0.0449 (11)	−0.0334 (8)	0.0380 (11)
C5	0.0411 (7)	0.1059 (15)	0.0622 (10)	−0.0024 (8)	−0.0149 (7)	0.0170 (10)
C6	0.0467 (7)	0.0562 (8)	0.0530 (7)	0.0033 (6)	−0.0174 (6)	−0.0028 (6)
C7	0.0331 (5)	0.0453 (6)	0.0352 (5)	−0.0070 (4)	−0.0118 (4)	0.0001 (4)
C8	0.0355 (5)	0.0482 (6)	0.0352 (5)	−0.0092 (4)	−0.0119 (4)	0.0043 (4)
C9	0.0548 (8)	0.0672 (9)	0.0376 (6)	−0.0123 (7)	−0.0193 (5)	0.0081 (6)
C10	0.0634 (9)	0.0818 (11)	0.0365 (6)	−0.0114 (8)	−0.0174 (6)	−0.0065 (7)
C11	0.0681 (10)	0.0686 (10)	0.0477 (8)	0.0010 (8)	−0.0175 (7)	−0.0198 (7)
C12	0.0559 (8)	0.0520 (7)	0.0486 (7)	0.0032 (6)	−0.0202 (6)	−0.0078 (6)
C13	0.0494 (7)	0.0433 (6)	0.0473 (7)	−0.0069 (5)	−0.0171 (5)	0.0061 (5)
C14	0.0797 (12)	0.0559 (9)	0.0628 (10)	0.0037 (8)	−0.0240 (8)	0.0138 (7)

### Geometric parameters (Å, °)

**Table:**

S1—O1	1.4246 (11)	C6—H6	0.9300
S1—O2	1.4280 (10)	C7—C12	1.3957 (19)
S1—N1	1.6274 (11)	C7—C8	1.4090 (17)
S1—C1	1.7555 (13)	C8—C9	1.3969 (17)
O3—C13	1.2260 (17)	C8—C13	1.4772 (19)
N1—C7	1.4049 (15)	C9—C10	1.374 (2)
N1—H1	0.8597	C9—H9	0.9300
C1—C2	1.3824 (18)	C10—C11	1.374 (3)
C1—C6	1.3834 (19)	C10—H10	0.9300
C2—C3	1.385 (2)	C11—C12	1.383 (2)
C2—H2	0.9300	C11—H11	0.9300
C3—C4	1.373 (3)	C12—H12	0.9300
C3—H3	0.9300	C13—C14	1.495 (2)
C4—C5	1.382 (3)	C14—H14A	0.9600
C4—H4	0.9300	C14—H14B	0.9600
C5—C6	1.380 (2)	C14—H14C	0.9600
C5—H5	0.9300		
			
O1—S1—O2	118.85 (7)	C12—C7—N1	121.74 (12)
O1—S1—N1	109.49 (6)	C12—C7—C8	119.64 (12)
O2—S1—N1	104.32 (6)	N1—C7—C8	118.58 (11)
O1—S1—C1	108.17 (7)	C9—C8—C7	117.87 (12)
O2—S1—C1	109.31 (6)	C9—C8—C13	119.87 (12)
N1—S1—C1	105.96 (6)	C7—C8—C13	122.26 (11)
C7—N1—S1	126.34 (9)	C10—C9—C8	122.23 (14)
C7—N1—H1	116.8	C10—C9—H9	118.9
S1—N1—H1	116.8	C8—C9—H9	118.9
C2—C1—C6	122.22 (13)	C11—C10—C9	119.21 (14)
C2—C1—S1	119.85 (11)	C11—C10—H10	120.4
C6—C1—S1	117.93 (10)	C9—C10—H10	120.4
C1—C2—C3	118.27 (15)	C10—C11—C12	120.77 (15)
C1—C2—H2	120.9	C10—C11—H11	119.6
C3—C2—H2	120.9	C12—C11—H11	119.6
C4—C3—C2	120.15 (16)	C11—C12—C7	120.27 (15)
C4—C3—H3	119.9	C11—C12—H12	119.9
C2—C3—H3	119.9	C7—C12—H12	119.9
C3—C4—C5	120.96 (15)	O3—C13—C8	122.15 (12)
C3—C4—H4	119.5	O3—C13—C14	118.48 (14)
C5—C4—H4	119.5	C8—C13—C14	119.37 (13)
C6—C5—C4	119.90 (18)	C13—C14—H14A	109.5
C6—C5—H5	120.0	C13—C14—H14B	109.5
C4—C5—H5	120.0	H14A—C14—H14B	109.5
C5—C6—C1	118.50 (16)	C13—C14—H14C	109.5
C5—C6—H6	120.7	H14A—C14—H14C	109.5
C1—C6—H6	120.7	H14B—C14—H14C	109.5
			
O1—S1—N1—C7	−57.47 (12)	S1—N1—C7—C12	29.02 (17)
O2—S1—N1—C7	174.32 (11)	S1—N1—C7—C8	−153.13 (10)
C1—S1—N1—C7	58.98 (12)	C12—C7—C8—C9	0.11 (18)
O1—S1—C1—C2	3.58 (12)	N1—C7—C8—C9	−177.78 (11)
O2—S1—C1—C2	134.36 (10)	C12—C7—C8—C13	179.40 (12)
N1—S1—C1—C2	−113.75 (10)	N1—C7—C8—C13	1.50 (17)
O1—S1—C1—C6	−175.46 (10)	C7—C8—C9—C10	−0.2 (2)
O2—S1—C1—C6	−44.68 (12)	C13—C8—C9—C10	−179.52 (14)
N1—S1—C1—C6	67.21 (11)	C8—C9—C10—C11	−0.2 (2)
C6—C1—C2—C3	0.07 (19)	C9—C10—C11—C12	0.7 (3)
S1—C1—C2—C3	−178.93 (10)	C10—C11—C12—C7	−0.8 (3)
C1—C2—C3—C4	−0.2 (2)	N1—C7—C12—C11	178.19 (13)
C2—C3—C4—C5	0.1 (2)	C8—C7—C12—C11	0.4 (2)
C3—C4—C5—C6	0.2 (3)	C9—C8—C13—O3	−178.77 (14)
C4—C5—C6—C1	−0.3 (2)	C7—C8—C13—O3	2.0 (2)
C2—C1—C6—C5	0.2 (2)	C9—C8—C13—C14	1.5 (2)
S1—C1—C6—C5	179.21 (12)	C7—C8—C13—C14	−177.78 (14)

### Hydrogen-bond geometry (Å, °)

**Table:**

*D*—H···*A*	*D*—H	H···*A*	*D*···*A*	*D*—H···*A*
N1—H1···O3	0.86	2.03	2.596 (2)	123
C2—H2···O1	0.93	2.52	2.893 (2)	104
C12—H12···O1	0.93	2.40	3.057 (2)	128
C11—H11···O1^i^	0.93	2.51	3.380 (2)	156
C14—H14C···Cg1^ii^	0.96	2.96	3.763 (2)	142

Symmetry codes: (i) −*x*, −*y*+2, −*z*; (ii) −*x*+1, −*y*+1, −*z*.
